# Label-Free Immunosensor Based on Polyaniline-Loaded MXene and Gold-Decorated β-Cyclodextrin for Efficient Detection of Carcinoembryonic Antigen

**DOI:** 10.3390/bios12080657

**Published:** 2022-08-19

**Authors:** Qi Wang, Huaqian Xin, Zhou Wang

**Affiliations:** Key Laboratory of Liquid-Solid Structural Evolution and Processing of Materials of Ministry of Education, School of Materials Science and Engineering, Shandong University, Jinan 250061, China

**Keywords:** electrochemical immunosensor, carcinoembryonic antigen, MXene, conductivity, antigen/antibody immobilization

## Abstract

Multiple strategies have been employed to improve the performance of label-free immunosensors, among which building highly conductive interfaces and introducing suitable biocompatible carriers for immobilizing antibodies or antigens are believed to be efficient in most cases. Inspired by this, a label-free immunosensor for carcinoembryonic antigen (CEA) detection was constructed by assembling AuNPs and β-CD (Au-β-CD) on the surface of FTO modified with PANI-decorated f-MXene (MXene@PANI). Driven by the high electron conductivity of MXene@PANI and the excellent capability of Au-β-CD for antibody immobilization, the BSA/anti-CEA/Au-β-CD/MXene@PANI/FTO immunosensor exhibits balanced performance towards CEA detection, with a practical linear range of 0.5–350 ng/mL and a low detection limit of 0.0429 ng/mL. Meanwhile, the proposed sensor presents satisfying selectivity, repeatability, and stability, as well as feasibility in clinic serum samples. This work would enlighten the prospective research on the alternative strategies in constructing advanced immunosensors.

## 1. Introduction

Cancer has ranked as a leading cause of alarming global deaths, with 19.3 million reported cases and 10.0 million deaths in 2020 [1]. The majority of cancer deaths are caused by the metastasis of malignant cancer cells to other organs through blood and the lymphatic system, thus making it essential to detect cancers at early stages before they become incurable [2]. However, the conventional detection techniques, oncological imaging and biopsy–cytology of specimens, are not applicable to common screening due to their drawbacks such as being tedious, expensive, and harmful to the body. The development of highly sensitive and effective diagnostic methods for the determination of cancer biomarkers is one of the most promising strategies for clinical diagnosis of cancers at early stages [3]. Carcinoembryonic antigen (CEA), with a normal concentration lower than 5 ng/mL in serum of healthy humans [4,5] and increased concentration induced by various cancers, is one of the most important clinical cancer biomarkers for the diagnosis of colon tumors, breast tumors, ovarian carcinoma, colorectal cancer, and cystadenocarcinoma [6,7,8,9].

The traditional CEA detection methods, including polymerase chain reaction (PCR) [10], enzyme-linked immunosorbent assay (ELISA) [11], and radio-immunoassays (RIAs) [12], usually require complicated equipment, radioactive chemicals, and tedious and time-consuming labeling processes. By contrast, the electrochemical immunosensor offers rapid and precise detection of CEA with low requirements of sample quantity at a relatively low cost [13,14,15,16]. In particular, the label-free immunosensor has attracted worldwide interest in research and application, since it eliminates the inherent disadvantages of the labeled immunosensor, such as the complex, time-consuming process and antigen–antibody inactivation during cumbersome steps. However, direct detection of CEA without signal amplification is extremely challenging, especially at an ultralow concentration. Multiple strategies have been employed to improve the performance of label-free immunosensors, including constructing high conductive sensing interfaces and designing suitable biocompatible carriers for immobilizing antibodies or antigens on the sensing surface [17,18,19,20].

Various nanomaterials have been applied to increase the conductivity of the electrode and amplify the electrochemical signal, among which MXene has achieved great successes owing to its unique properties, such as high electron transfer capacity, high specific surface area, biocompatibility, abundant surface chemical groups, and hydrophilicity [21,22]. Kumar et al. developed a high-sensitivity immunosensor by using biofunctionalized Ti_3_C_2_-MXene [23]. Liu constructed MXene-Ti_3_C_2_T_x_-based screen-printed electrodes and assembled a dialysis microfluidic sensor, which was successfully applied to hemodialysis and enabled continuous detection of urea, uric acid, and creatinine levels in human blood [24]. However, similar to other 2D nanomaterials, the electrochemical performance of MXene-based composites is prone to be affected by their self-stacking nature due to strong van der Waals interactions between adjacent nanosheets. To overcome this weakness, many studies have focused on the strategy of introducing interlayer nanomaterials [25,26,27]. On the other hand, the –F functional group on MXene is not ideal for the immobilization of antibodies or antigens. Thus, designing a new carrier that is capable of facilitating immobilization of antibodies as well as amplifying the electrochemical signal is very important.

In this work, the composite of polyaniline (PANI)-loaded few-layered MXene (f-MXene) was firstly decorated onto the fluorine-doped indium tin oxide glass (FTO) through the electrophoretic deposition (EPD) method, which plays the role of improving the conductivity, electrocatalytic activity, and sensitivity of the immunosensor. Then, gold nanoparticles (AuNPs)-decorated β-cyclodextrin (β-CDs) were chosen for immobilizing CEA-antibody and assembled on the MXene@PANI layer through the direct electrodeposition (ED) method. Specifically, Au helps the immobilization of CEA-antibody via the strong interaction between Au and sulfhydryl group on the antibody [28], while β-CDs improve the immobilization through their unique toroidal structure with a hydrophobic inner cavity and a hydrophilic outer side [29], which enables selective binding with several organic molecules in the cavities and forms stable host–host inclusion complexes. Therefore, a label-free electrochemical immunosensor based on FTO that is modified with MXene@PANI and immobilized with Au-β-CDs-conjugated CEA-antibody was reported first, and it was termed Au-β-CD/MXene@PANI-antiCEA.

## 2. Materials and Methods

### 2.1. Reagents and Apparatus

All reagents were purchased from suppliers and were used directly without purification. The CEA and CEA antibody were purchased from Linc-Bio Science Co., Ltd. (Shanghai, China). MAX (Ti_3_AlC_2_) power was purchased from 11 Technology Co., Ltd. (Jilin, China). Hydrochloric acid (HCl), lithium fluoride (LiF), β-Cyclodextrin (β-CD), and bovine serum albumin (BSA, 96–99%) were purchased from Macklin Biochemical Co., Ltd. (Shanghai, China). Aniline, ethanol (C_2_H_6_O), ammonium persulfate ([NH_4_]_2_S_2_O_8_) (APS), aniline monomer (ANI), chloroauric acid trihydrate (HAuCl_4_·3H_2_O), potassium hexacyanoferrate(II) (K_4_[Fe(CN)_6_]), potassium ferricyanide (K_3_[Fe(CN)_6_]), and potassium chloride (KCl) were purchased from Sinopharm Chemical Reagent Co., Ltd. (Beijing, China). Phosphate-buffered saline (PBS) was obtained by compounding the solution of Na_2_HPO_4_·2H_2_O and NaH_2_PO_4_·12H_2_O as the electrolyte in the process of electrochemical measurements. Fluorine-doped tin oxide (FTO) was purchased from Kaivo Optoelectronic Technology Co., Ltd. (Zhuhai, China).

A field emission high-temperature scanning electron microscope (SEM, JEOL JSM-7800F from Tokyo, Japan) equipped with an energy-dispersive spectrometer (EDS, Oxford Instruments Xmax-80 from Oxford, UK) was applied to analyze the surface morphologies and composition. The interior structure of few-layered MXene was investigated by transmission electron microscopy (TEM, JEOL JEM-2100F from Tokyo, Japan) operated at 200 kV. The crystalline phases of products were characterized by an X-ray diffractometer (XRD) on Bruker DMAX-2500PC (Massachusetts, NE, USA) with Cu Kα radiation (λ = 0.15406 nm). The chemical valence states and surface compositions were studied via X-ray photoelectron spectroscopy (XPS) spectra recorded by Kratos AXIS Supra (Manchester, UK) equipped with a monochromatism Al Kα. Contact angle measuring instrument (DSA100S) from Krüs Scientific Instruments Shanghai Co., Ltd. (Shanghai, China) was employed to evaluate the hydrophobicity of the electrodes.

### 2.2. Preparation Processes of Immunosensor Electrode

**Scheme 1:**
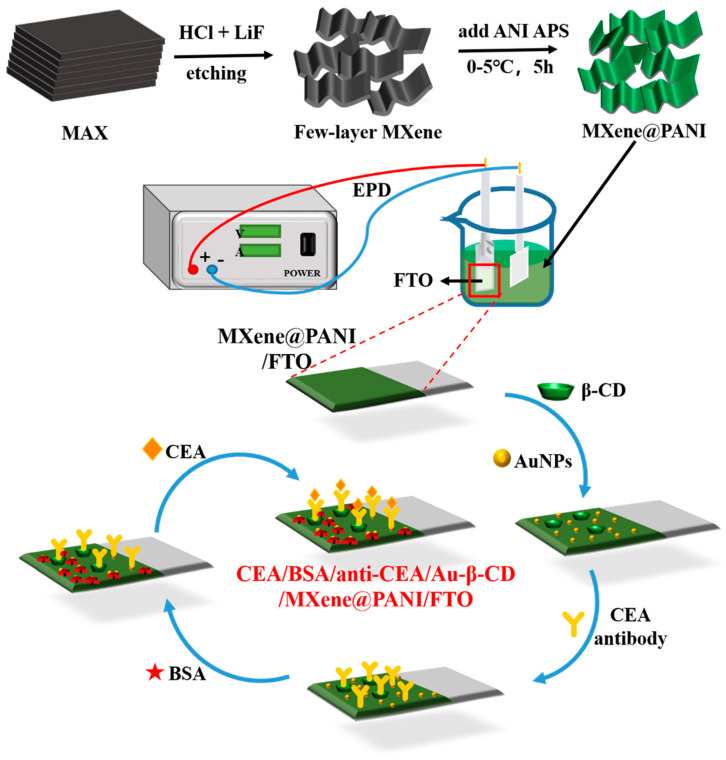
Schematic diagram of the electrochemical immunosensor fabrication steps for label-free detection of CEA.

The fabrication procedure of the electrochemical immunosensor is outlined in Scheme 1.

Preparation of f-MXene: Typically, 2 g LiF was dissolved in 9 M HCl solution to obtain a homogeneous solution. Then, 2 g Ti_3_AlC_2_ powder was slowly added into the above solution and kept under continuous stirring at a speed of 400 rpm for 24 h with a set temperature of 35 °C. Then, the obtained mixture was centrifuged at a speed of 3500 rpm and washed several times by distilled water until pH = 5. In the next step, the residual in the centrifuge tube was added into ethanol and sonicated for 1 h. After the ultrasonic treatment, the upper suspension of the solution was collected. At last, the suspension was sonicated for another 20 min and centrifuged at a speed of 3500 rpm, and the supernatant which contained few-layered MXene (f-MXene) was collected. More f-MXene can be obtained by repeating the ultrasonication and centrifugation processes.

Preparation of MXene@PANI: The experimental procedure is in reference to a previously reported method [30]. Firstly, 0.2 g f-MXene was dispersed into 30 mL of 1 M HCl solution under nitrogen protection for full ultrasound for 1 h. Secondly, 100 μL ANI was added into the above mixture and magnetically stirred for 30 min at 0–5 °C. Then, 0.135 g APS powder was dispersed in 8 mL of 1 M HCl to prepare the oxidation solution. The oxidant solution was slowly added into the MXene/HCl/ANI suspension in 30 min. Then, the mixture was magnetically stirred for 5 h at 0–5 °C. The obtained suspension was washed by DI water to separate the nanocomposite from HCl.

Construction of Au-β-CD/MXene@PANI electrode: Before the formal procedures, the FTO substrates were cleaned with HCl, deionized water, acetone, and ethanol and then dried in nitrogen atmosphere. The MXene@PANI nanocomposites were loaded on FTO through an EPD process in MXene@PANI suspension. The voltage of the EPD process was optimized (detailed in Section 3.3). After being dried in air, the one-step electrodeposition method was applied to load AuNPs and β-CD on the above electrode. Specifically, the deposition was performed in a stirred 0.1 M PBS containing 1.25 mM HAuCl_4_ and 0.15 mg/mL β-CD at a fixed potential of −1.4 V. The deposition time was also optimized (detailed in Section 3.3). Finally, the Au-β-CD/MXene@PANI electrode was obtained.

Construction of the immunosensor: Firstly, the Au-β-CD/MXene@PANI electrode was incubated with anti-CEA (10 μg/mL, 20 μL) and dried at 4 °C. The incubation time was optimized (detailed in Section 3.3). After being washed with PBS, 15 μL of 1 wt% BSA solution was dropped onto the surface of the anti-CEA/Au-β-CD/MXene@PANI/FTO electrode and incubated for 40 min to eliminate nonspecific protein-binding sites. BSA was loaded in order to close the specific site and avoid any impact on the accuracy of the experimental results. Subsequently, the BSA/anti-CEA/Au-β-CD/MXene@PANI/FTO was washed with PBS and incubated in different concentrations of CEA antigen for 40 min at 4 °C. Finally, the CEA/BSA/anti-CEA/Au-β-CD/MXene@PANI/FTO was washed thoroughly by PBS to remove nonspecific adsorption of antigen-binding molecules. The electrode at every step was cleaned by 0.01 M PBS (pH = 7.4). The modified CEA immunosensor was stored in a refrigerator for further measurement.

### 2.3. Electrochemical Measurements

All electrochemical measurements were performed on the Ivium Vertex One EIS electrochemical workstation via a conventional three-electrode system with an FTO (active area 1 × 1 cm^2^), Ag/AgCl electrode (3.5 M KCl), and a platinum foil electrode as working, reference, and counter electrode, respectively. The CV measurement was carried out in 0.01 M PBS containing 5 mM [Fe(CN)_6_]^3−^/[Fe(CN)_6_]^4−^ in the range of −0.1 to +0.7 V. EIS was also performed in the same solution. The amplitude was 10 mV and the frequency ranged from 0.1 Hz to 100 kHz.

## 3. Results and Discussion

### 3.1. Materials Characterization

**Figure 1:**
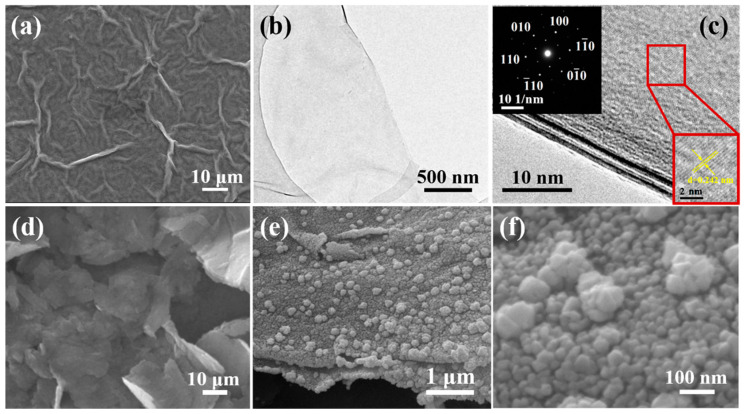
(**a**) SEM, (**b**) TEM, and (**c**) HRTEM images of f-MXene. SEM images of (**d**) MXene@PANI and (**e**,**f**) Au-β-CD/MXene@PANI.

The morphology of nanomaterials was investigated to study the morphology evolution at different fabrication stages. As shown in the scanning electron microscope (SEM) image (Figure 1a), large-size (over 100 μm) f-MXene with characteristic wrinkled surface was successfully fabricated. In order to explore the detailed morphology of the f-MXene, transmission electron microscopy (TEM) was conducted and is shown in Figure 1b,c. The low contrast of the sample in Figure 1b verifies the thinness of the obtained f-MXene. According to the high-resolution TEM (HRTEM) image in Figure 1c, f-MXene presents a determined interplanar spacing of 0.242 nm, which corresponds to the {104} crystal plane group. Moreover, as demonstrated in the selective-area electron diffraction (SAED) pattern (the inset in Figure 1c), the prepared f-MXene has a single crystallinity structure with hexagonal symmetry. In order to confirm the effective exfoliation from MAX to f-MXene, the XRD patterns of both samples were recorded (Figure S1). The XRD pattern of MAX matches well with the standard card JCPDS No. 052-0875. The f-MXene obtained by etching and stripping exhibits quite a different pattern. Firstly, the disappeared peak at 2θ = 39°, which corresponds to the (104) plane of MAX, indicates the complete removal of the aluminum element by etching. Secondly, the position of the (002) diffraction peak decreases from 9.73° to 6.58°, which verifies that an increase of 0.435 nm in the interlayer spacing *d* of (002) crystal plane was induced by etching. Finally, other characteristic peaks of MAX become very weak in f-MXene, which can be ascribed to the disruption of the long-range lattice periodicity in the few-layer structure [31].

As shown in Figure 1d, after being compounded with PANI, the complex shows a broken lamellar structure with a size of about 2~3 μm. It is noteworthy that the thickness of nanosheets increased significantly with only slight agglomeration, which proves that PANI was successfully and uniformly loaded on f-MXene. Further deposition of AuNPs and β-CD induced obvious change in morphology (Figure 1e,f). Although the lamellar structure maintained, the surface turned rough and was homogeneously covered by closely packed nanoparticles with a diameter of 60~70 nm, indicating the successful deposition of AuNPs and β-CD.

**Figure 2:**
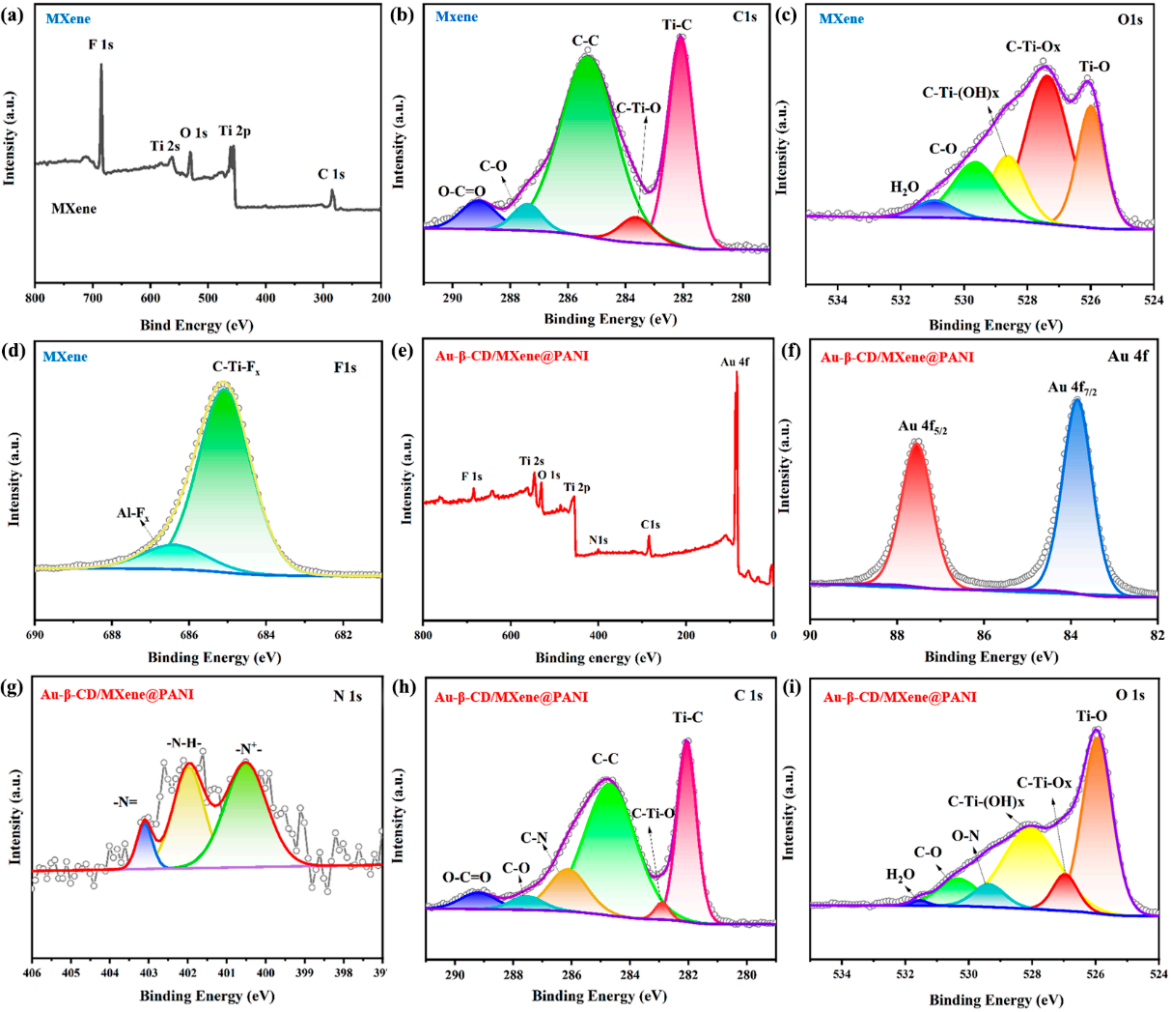
XPS spectra of f-MXene: (**a**) survey, (**b**) C 1s, (**c**) O 1s, and (**d**) F 1s. XPS spectra of Au-β-CD/MXene@PANI: (**e**) survey, (**f**) Au 4f, (**g**) N 1s, (**h**) C 1s, and (**i**) O 1s.

Besides direct observation through high-resolution imaging instruments, the effect of each component on the electron structure was studied through XPS test. Figure 2a shows the wide XPS survey spectrum of f-MXene, which is mainly composed of C, Ti, O, and F elements. The C 1s spectrum of f-MXene (Figure 2b) can be decomposed into five peaks located at 282.1, 283.7, 284.8, 287.4, and 289 eV, corresponding to C–Ti, C–Ti–O, C–C, C–O, and O–C=O, respectively [32]. The O 1s spectrum shown in Figure 2c can be deconvoluted into five individual peaks at 526.5, 527.8, 528.5, 529.5, and 531.2 eV, which can be ascribed to Ti–O, C–Ti–Ox, C–Ti–(OH)x, C–O, and surface-adsorbed H_2_O, respectively. Among them, the chemical binding of Ti–O and C–Ti–Ox indicates the presence of surface oxidization in f-MXene, while C–Ti–(OH)x can be attributed to the capping hydroxyl produced during the acid-etching process [33,34]. The asymmetric shape of the F 1s spectrum (Figure 2d) indicates the diverse bonding situation of F atoms in f-MXene, which can be deconvoluted into two peaks, corresponding to C–Ti–F*_x_* and Al-F*_x_*, respectively. The C–Ti–F*_x_* bond accounts for the most part, verifying the abundant –F functional group on the surface. The weak signal of Al–F*_x_* testifies the low Al residual in f-MXene [35]. 

The XPS spectra of Au-β-CD/MXene@PANI were also recorded to study the effect of subsequent loading of PANI, AuNPs, and β-CD. The survey spectrum in Figure 2e differs obviously from pure f-MXene (Figure 2a) in two aspects: Firstly, the new characteristic peaks of nitrogen (N) and gold (Au), which corroborate the successful modification of PANI and AuNPs; secondly, the sharply declining peak intensity of F element, which can be attributed to the limited detection depth of XPS. The high-resolution Au 4f spectrum (Figure 2f) exhibits two characteristic peaks centered at 83.8 eV (4f_7/2_) and 87.5 eV (4f_5/2_) with an energy gap of 3.7 eV, which confirms the presence of Au^0^ in Au-β-CD/MXene@PANI [36]. The XPS signal of N element is weak but identifiable in the high-resolution N 1s spectrum (Figure 2g), which can be fitted into three peaks, centered at 400.5 eV, 402.0 eV, and 403.1 eV, respectively. These peaks correspond to three types of nitrogen functional groups: quinoid imine (–N=), benzenoid amine (–NH–), and cationic amine (–N^+^–) [37], which is in accordance with the chemical bonding of N in PANI, confirming the successful loading of PANI. The high-resolution C 1s and O 1s spectra (Figure 2h,i) of Au-β-CD/MXene@PANI are identical with that of pure f-MXene, except for one emerging peak in each spectrum. The appearance of C–N bonding at 286.3 eV in C 1s and O–N bonding at 529.5 eV in O 1s further evidences the existence of PANI.

Biological processes and their associated biological functions are closely related to hydrophilic interactions, making it important to regulate the hydrophilicity of interfaces for substrates required to be loaded with biomolecules. In order to test the effect of the loading components on surface-wetting property, the contact angle measurement was carried out on bare FTO, Au-β-CD/MXene@PANI/FTO, anti-CEA/Au-β-CD/MXene@PANI/FTO, and BSA/anti-CEA/Au-β-CD/MXene@PANI/FTO (Figure S2). Compared with bare FTO (32.8°), the lower contact angle of Au-β-CD/MXene@PANI/FTO (26.0°) demonstrates its improved hydrophilicity, which is in favor of subsequent protein immobilization. The contact angle of anti-CEA/Au-β-CD/MXene@PANI/FTO (12.2°) and BSA/anti-CEA/Au-β-CD/MXene@PANI/FTO (3.5°) kept the decreasing trend, indicating the successful loading of antibody and BSA on the electrode surface. The excellent hydrophilicity of the electrodes is beneficial for elevating the CEA detection performance of the immunosensor.

### 3.2. Electrochemical Characterization of Immunosensor

**Figure 3:**
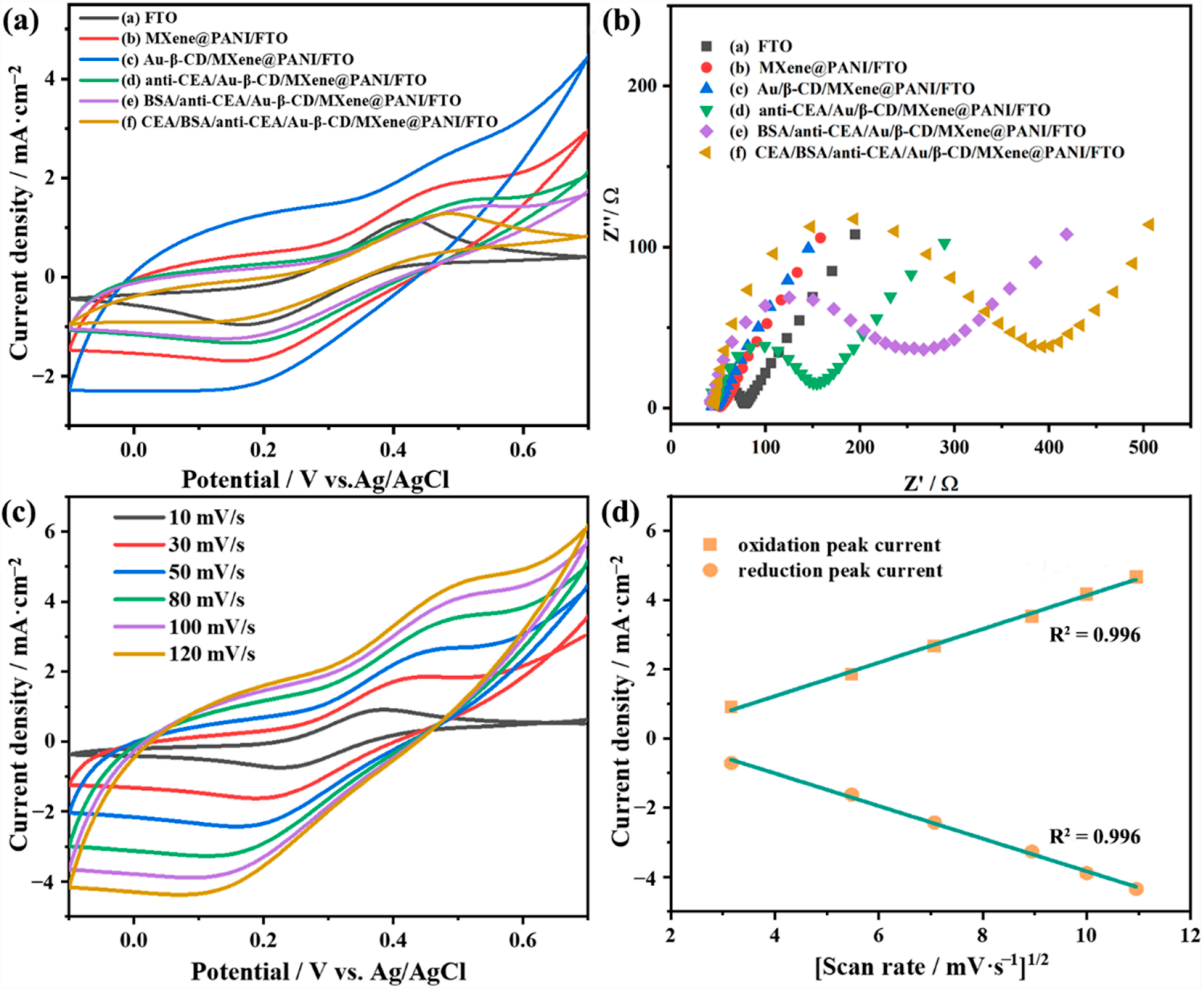
(**a**) Cyclic voltammogram and (**b**) EIS spectra of electrodes at different fabrication steps. (**c**) Cyclic voltammogram and (**d**) Randles–Ševčík plot of Au-β-CD/MXene@PANI/FTO at different scan rates from 10 to 120 mVs^−1^.

In order to probe into the electrochemical properties of the constructed immunosensor, CV and EIS techniques were applied in monitoring the electrochemical changes on the electrode obtained at different fabrication stages. As shown in Figure 3a, the CV curves were utilized to characterize the fabrication process of the immunosensor in 0.01 M PBS containing 5.0 mM [Fe(CN)_6_]^3−/4−^. Compared to bare FTO (curve a), the redox peak of the MXene@PANI/FTO electrode (curve b) obtained increased intensity due to the improved electrochemical properties, which benefit from the high conductivity and redox ability of MXene@PANI complex. Further deposition of AuNPs and β-CD also induces an obvious enhancement in peak current, accompanied by the potential gap (ΔE) between the oxidation peak and reduction peak decreasing due to the unique property of gold nanoparticles to promote electron transfer. Subsequently, when anti-CEA (curve d), BSA (curve e), and CEA (curve f) were sequentially modified onto the immunoelectrode, the peak current gradually decreased and the potential difference (ΔE) of the oxidation/reduction peak gradually increased, which can be ascribed to the hindered electron transfer induced by protein molecules on the electrode surface. The above change in law of peak intensity accords with the immunosensing mechanism, indicating the successful construction of the immunosensor.

Nyquist plots were recorded between neighboring fabrication steps of the immunosensor to investigate the role of each layer in the evolution of electron transfer property, as shown in Figure 3b and Figure S3. When modified with MXene@PANI (curve b), the electron transfer resistance (R_ct_) decreased from 81 Ω (bare FTO, curve a) to 57 Ω. After AuNPs and β-CD were electrodeposited on the electrode (curve c), the R_ct_ further decreased to 47 Ω, owing to the excellent electrical transport properties of AuNPs. Subsequently, anti-CEA/Au-β-CD/MXene@PANI/FTO (curve d) showed clearly increased R_ct_ value of 158 Ω when anti-CEA was assembled on the Au-β-CD/MXene@PANI/FTO. The loaded anti-CEA hindered the electron transfer on the electrode surface. When bovine serum protein (BSA) was immobilized on the surface of anti-CEA/Au-β-CD/MXene@PANI/FTO, the R_ct_ further increased to 297 Ω (curve e) since the protein molecules blocked the electron transfer. At last, R_ct_ further increased to 392 Ω after the addition of CEA (curve f). According to the impedance results, the evolution of R_ct_ at each stage of electrode construction and immunosensing testing is inconsistent with the working mechanism of immunosensing, which further verifies the successful construction of the label-free CEA electrochemical immunosensor.

The interface dynamic behaviors of Au-β-CD/MXene@PANI/FTO in 0.01 M PBS containing 5 mM [Fe (CN)_6_]^3−/4−^ were investigated by a series of different scan rates from 10 to 120 mV·s^−1^. As shown in Figure 3c, the current density of the anode (I_pa_) and cathode (I_pc_) increases gradually and the peak potentials of the anode and cathode shift to the edge of the voltage window with the growing scan rate. The peak current intensities derived at different scant rates were recorded and fitted into the coordinate, in which the horizontal axis denotes the square root of the scan rates while the vertical axis represents the value of peak current corresponding to the subtracted background at different sweep rates (subtracted background peak current = absolute value − background value at the tangent). As shown in Figure 3d, the linear fit reveals that both I_pa_ and I_pc_ increase linearly with the square root of the scan rate, which indicates that the electrochemical reaction is a diffusion-controlled process. The linear equations are as follows:

I_pc_ = [0.352 mA × (scan rate[mV s^−1^])^1/2^] − 0.556 mA, R^2^ = 0.99 (1)

I_pa_ = [−0.331 mA × (scan rate[mV s^−1^])^1/2^] + 0.604 mA, R^2^ = 0.99 (2)

Based on the above equations, the diffusion coefficient of the redox probe for the Au-β-CD/MXene@PANI/FTO electrode was calculated to be 4.823 × 10^−18^ cm^2^/s with an electrochemically active specific surface area (EASA) of 0.0106 cm^2^ according to the Randles–Ševčík equation. 

### 3.3. Optimization of Experimental Conditions

In order to achieve the best performance, one key experimental condition in each fabrication step, including the voltage of EPD process, the deposition time of electrodeposition process, the incubation time of anti-CEA, and the pH of electrolyte for electrochemical evaluation, was selected and optimized by conducting cyclic voltammetry (CV) tests in 0.01 M PBS containing 5 mM [Fe(CN)_6_]^3−/4−^.

**Figure 4:**
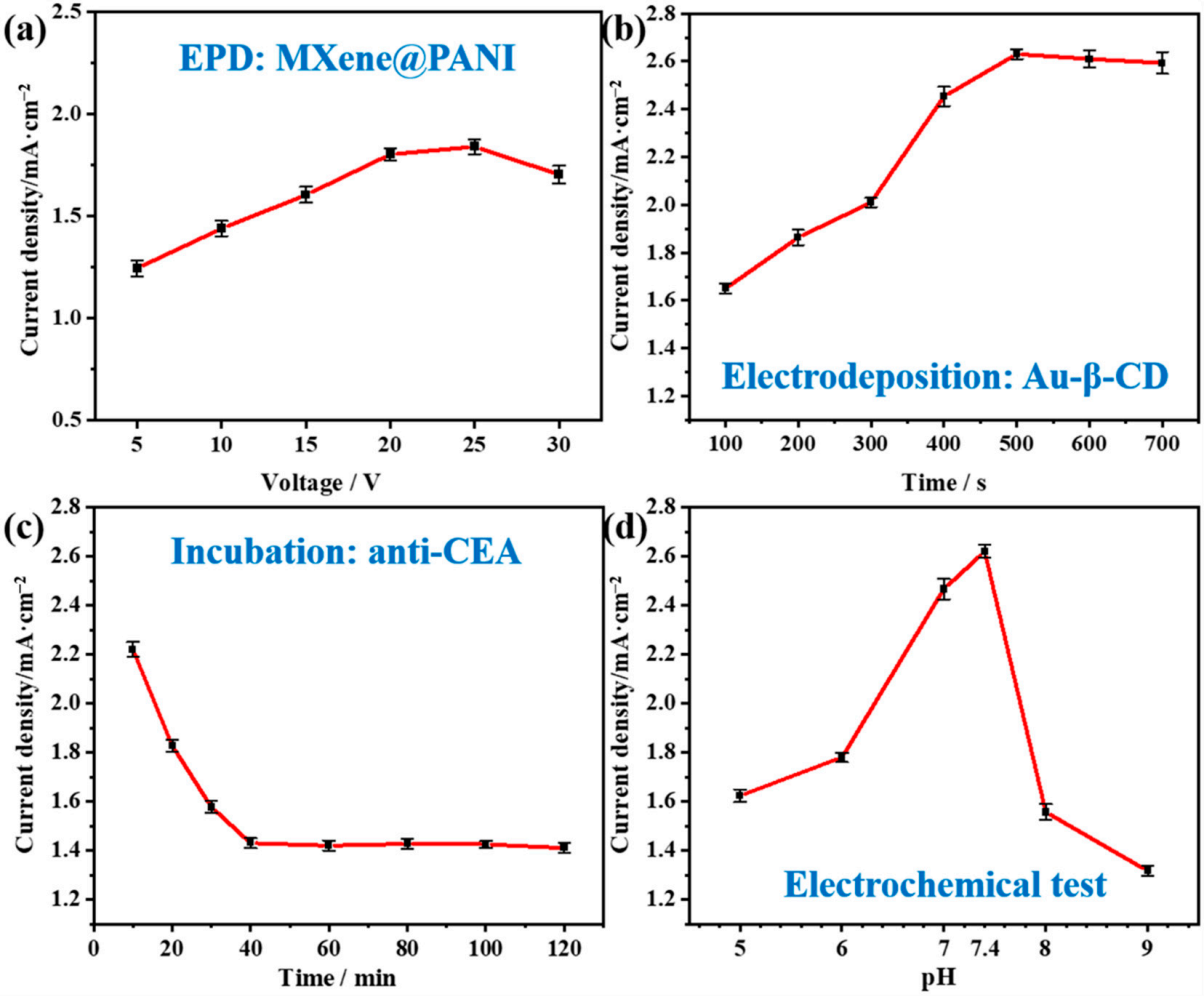
Current response to the variation of (**a**) voltage of EPD process, (**b**) deposition time of electrodeposition process, (**c**) incubation time of anti-CEA, and (**d**) pH of the electrolyte for electrochemical evaluation.

The voltage applied in the EPD of MXene@PANI should be strictly disciplined, since high voltage may induce the oxidation of MXene and lead to conductivity attenuation [38]. The relationship between the current response and the EPD voltage in a range from 5 V to 30 V is shown in Figure 4a. A clear enhancement of the response was observed with the increasing voltage from 5 V to 20 V, followed by a slightly increased current at 25 V. Further elevation of voltage to 30 V induced larger internal stress, which produced some warped area on the surface of the electrode and broke the contact of active materials with FTO substrate, resulting in a decreased current response. Considering the identical response obtained at 20 V and 25 V, 20 V was chosen as the optimum voltage for EPD to avoid the possible oxidation of MXene at higher voltage.

The electrodeposition time of Au-β-CD was evaluated within the range of 100~700 s since the modification of Au-β-CD directly decides the subsequent loading of anti-CEA, which has a close relationship with the CEA detection performance. As shown in Figure 4b, the signal response gradually increased with the prolonged time until reaching 500 s, and the signal remained almost constant afterward. Thus, the electrodeposition time of 500 s was considered to be optimum for the immunosensor construction in this work.

To enhance the sensitivity of the immunosensor, the incubation time of the antibody on the electrode surface was further optimized, and the results are shown in Figure 4c. The current intensity decreased greatly with the increasing incubation time and remained almost constant after 40 min, owing to the saturated amount of the antibody on the modified electrode surface. Thus, 40 min was considered to be the optimum incubation time in the subsequent study.

The pH value plays a key role in evaluating the performance of the immunosensor for two reasons. Firstly, highly acidic and alkaline electrolytes could affect the bioactivity of the immobilized antibody and cause the antibody denaturation. Secondly, the real application of one immunosensor is directly limited by its applicable pH environment, especially in real-time monitoring application scenarios. As shown in Figure 4d, the highest response was achieved at pH of 7.4, which is the normal pH value in the human body, verifying the immense potential of our proposed immunosensor in medical applications.

### 3.4. CEA Sensing Performance of the Immunosensor

**Figure 5:**
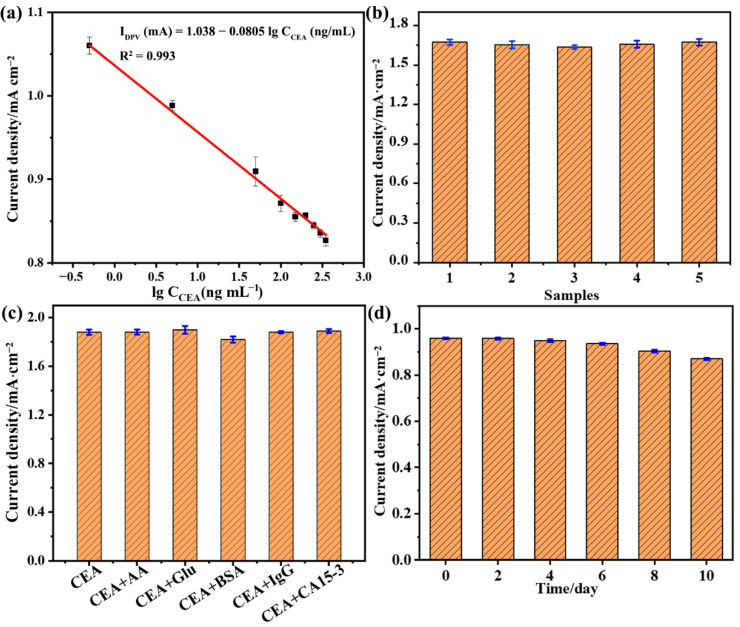
(**a**) Calibration plots of the electrochemical immunosensor for CEA detection. (**b**) Current response of five immunosensors to 100 ng/mL CEA. (**c**) Current response of the immunosensor to pure CEA and CEA mixed with 100 ng/mL of other interference substances. (**d**) The current density response to 5 ng/mL CEA after different durations.

The CEA sensing performance of the constructed BSA/anti-CEA/Au-β-CD/MXene@PANI/FTO immunosensor was evaluated using the differential pulse voltammetry (DPV) method. The DPV curves were recorded by performing the test in a group of 0.01 M PBS solution (with 5 mM [Fe (CN)_6_]^3−/4−^ as the probe) containing different amounts of CEA (0~350 ng/mL). As shown in Figure S4, the peak current responds evidently and exhibits a decreasing trend towards the growing concentration of CEA. The decaying peak current is attributed to the impeded electron transport on the electrode surface after the CEA antigen is trapped by CEA. The calibration curve shown in Figure 5a evidences the well-fitted linear relationship between the CEA concentration, in logarithm, and current density, testifying its ratiometric CEA-detection capability. More specifically, the linear regression equation of our BSA/anti-CEA/Au-β-CD/MXene@PANI/FTO immunosensor is calculated to be I_DPV_ (mA) = 1.038–0.0805 lg C_CEA_ (ng/mL) (R^2^ = 0.993) with a wide linear detection range of 0.5~350 ng/mL and a low detection limit of 0.0429 ng/mL at a signal-to-noise ratio of 3.

In order to obtain an objective evaluation of the proposed sensor in our work, the typical sensing-performance indicators (linear range and detection limit) were compared with other CEA immunosensors reported in previous studies [3,39,40,41,42,43,44,45,46,47]. Considering that the normal concentration of CEA in serum of healthy humans is lower than 5 ng/mL [4,5], our Au-β-CD/MXene@PANI/FTO sensor is significant in the balanced performance with practical linear range (0.5–350 ng/mL) and impressive detection limit (0.0429 ng/mL), as verified by the summarized data in Table 1. The superior capability of our sensor in quantitative detection of high CEA concentration enables applications in monitoring the development of cancer in patients suffering from mid- or advanced-stage cancer. Meanwhile, the relatively low detection limit is applicable for cancer screening.

The above-mentioned performance indicators were commonly obtained with the best sample in ideal condition. However, many other factors, such as the consistency between different batches, the disturbance from other substances, the stability in long-term storage, etc., must be considered in real applications. Inspired by the real requirements, three top essential application indicators selectivity, repeatability, and stability, were evaluated by recording the peak current density in diverse situations. Firstly, the investigation of reproducibility was carried out on five electrodes prepared independently following the same procedure. The current intensity of five electrodes was measured in the presence of 100 ng/mL CEA, and a satisfied relative standard deviation (RSD) of 2.61% was achieved (Figure 5b). Secondly, the selectivity of the constructed electrodes was investigated by performing the DPV test in electrolytes containing 100 ng/mL of CEA mixed with other interfering substances. Since the sensor is designed to detect CEA in human serum, several types of common biomolecules and typical cancer markers, including ascorbic acid (AA), glucose (Glu), bovine serum protein (BSA), human immunoglobulin G (IgG), and cancer antigen 15-3 (CA15-3), were selected as the interferents. As shown in Figure 5c, the current intensity of the DPV curves almost stays steady in various electrolytes. The injection of interferents engenders no evident response, indicating the high selectivity of our sensor. According to the previous research, MXene is susceptible to being oxidized by moisture and oxygen in the air, forming a white turbid titanium dioxide colloidal solution. Since MXene is the main component of our sensor, it is crucial to pay special attention to the stability in long-term storage. The storage stability of the immunosensors was checked by storing them at 4 °C in general atmospheric environment and recording their current response periodically in the presence of CEA 5 ng/mL every two days. The histogram, shown in Figure 5d, verifies a retention of 90.6% of its initial value after 10 days, indicating its high storage stability. In summary, the above DPV tests demonstrate that our BSA/anti-CEA/Au-β-CD/MXene@PANI/FTO immunosensor possesses good selectivity, excellent reproducibility, and high stability.

Furthermore, the sensing performance of our immunosensor in real human serum was investigated to evaluate its feasibility in practical applications. Briefly, healthy human serum samples were diluted 10 times, then standard concentrations of CEA (15 ng/mL, 25 ng/mL, 75 ng/mL, 200 ng/mL) were added into the diluted serum. Then, the produced current signal was recorded by DPV test, through which the concentration of CEA was detected and calculated according to a regression equation obtained from the linear relationship in Figure 5a. The results are listed in Table 2, and the calculated recovery ratios of our BSA/anti-CEA/Au-β-CD/MXene@PANI/FTO immunosensor in clinic serum samples range from 97.52% to 103.98%, demonstrating its good detection accuracy of CEA in real biological samples and its potential for large-scale applications.

## 4. Conclusions

In this work, an electrochemical immunosensor for label-free detection of CEA was designed and constructed based on immobilizing CEA antibody conjugated with AuNPs and β-CD (anti-CEA/Au-β-CD) on the surface of FTO modified with PANI-decorated f-MXene (MXene@PANI). The differential pulse voltammetry (DPV) method was applied to monitor the concentration of CEA. The key fabrication indicators of our immunosensor are optimized with a wide linear range of 0.5~350 ng/mL and a low detection limit of 0.0429 ng/mL. The feasibility of the immunosensor for CEA detection in human serum samples was also verified by a good correspondence between values measured by the immunosensor and those actually added. The excellent sensing performance can be attributed to the synergistic effect of the components in the constructed sensor: (i) the stable composites of MXene@PANI through Ti–N bonding effectively avoid the agglomeration and oxidation of f-MXene, achieving a uniform electrode substrate with large specific surface area, abundant active sites, high electrical conductivity, and fast electron transfer efficiency; (ii) AuNPs and β-CD form a stable host–guest inclusion complex through organic, inorganic, and biologic guest molecules, which further enhances the electrical conductivity, hydrophilicity, and stability of the composite electrode and realizes efficient loading of anti-CEA, hence improving the sensitivity of the immunosensor for CEA detection. Our investigation on the construction and electrochemical properties of the newly developed immunosensor provides a necessary experimental and theoretical foundation for the future design of convenient, low-cost, and high-performance sensors.

## Figures and Tables

**Table 1:** Comparison of linear range and detection limit of various CEA immunosensors.

	Assay Principle	Linear Range (ng/mL)	Detection Limit (ng/mL)	Ref.
Au-β-CD/MXene@PANI/FTO	DPV	0.5–350	0.0429	This work
3D-Graphene/pDA/HRP/Ab	DPV	0.1–750.0	0.09	[39]
Water-soluble graphene sheet/PB-chitosan/Nanoporous gold/GCE	CV	0.02–14	0.0631	[40]
Ag/MoS_2_/rGO/GCE	i-t	0.0001–100	1.6 × 10^−6^	[41]
AuPdCu/N-GQDs@PS/GCE	i-t	0.01–50	3.3 × 10^−6^	[42]
AuNPs/polyaniline/COOH-MWCNTs/GCE	DPV	1.66 × 10^−6^–1.3	0.5 × 10^−3^	[43]
anti-CEA/rGO/GCE	CV	0.1–5	0.05	[44]
SPCE/GNP-MnO_2_/Fe_3_O_4_@Au	LSV	0.001–100	0.10 × 10^−3^	[3]
AuNPs/CNOs/SWCNTs/CS/GCE	SWV	1.0 × 10^−4^–400	0.1	[7]
Rh@Pd NDs /MWCNTs-SO_3_H	DPV	2.5 × 10^−5^–100	8.3 × 10^−6^	[45]
ITO/WO_3_@BiOI@CdS	PEC	0.01–50	3.2 × 10^−6^	[46]
PtPd/N-GQDs@Au	i-t	5.0 × 10^−6^–50	2 × 10^−6^	[47]

**Table 2:** Analytical results for the determination of CEA in clinic serum samples.

Sample No.	Added CEA (ng/mL)	Founded CEA (ng/mL)	Recovery (%)	RSD (%, n = 5)
1	15	14.6	97.52	2.74
2	25	25.1	100.24	1.18
3	75	75.9	101.32	0.75
4	200	207.9	103.98	2.44

## Data Availability

Not applicable.

## Supplementary Materials

The following supporting information can be downloaded at: https://www.mdpi.com/article/10.3390/bios12080657/s1, Figure S1: XRD patterns of MAX and f-MXene; Figure S2: Water contact angle of different electrode (a) FTO, (b)Au-β-CD/MXene@PANI/FTO, (c) anti-CEA/Au-β-CD/MXene@PANI/FTO, and (d) BSA/anti-CEA/Au-β-CD/MXene@PANI/FTO; Figure S3: Amplification of electrochemical impedance curves of different modified electrodes; Figure S4: DPV responses for label-free immunosensor to CEA at the concentrations of 0, 0.5, 5, 50, 150, 200, 250, 300, and 350 ng/mL in 0.01M PBS containing 5.0 mM [Fe (CN)_6_]^3−/4−^.
