# Supplementary material for: Label-Free Immunosensor Based on Polyaniline-Loaded MXene and Gold-Decorated β-Cyclodextrin for Efficient Detection of Carcinoembryonic Antigen
Source: Biosensors (Basel). 2022 Aug 19;12(8):657. doi: 10.3390/bios12080657 (PMC9405772; doi:10.3390/bios12080657)
Supplement: Supplementary file 1 [file biosensors-12-00657-s001.zip › biosensors-1855421-SI.pdf]

biosensors

MDPI

Supplementary Materials:Label-Free Immunosensor Based on Polyaniline-Loaded MXene and Gold-Decorated β-Cyclodextrin for Efficient Detection of Carcinoembryonic Antigen

Qi Wang, Huaqian Xin and Zhou Wang *

Key Laboratory of Liquid-Solid Structural Evolution and Processing of Materials of Ministry of Education, School of Materials Science and Engineering, Shandong University, Jinan 250061, China; wangqi1016@sdu.edu.cn (Q.W.); 15665812750@163.com (H.X.)

* Correspondence: wangzhou@sdu.edu.cn

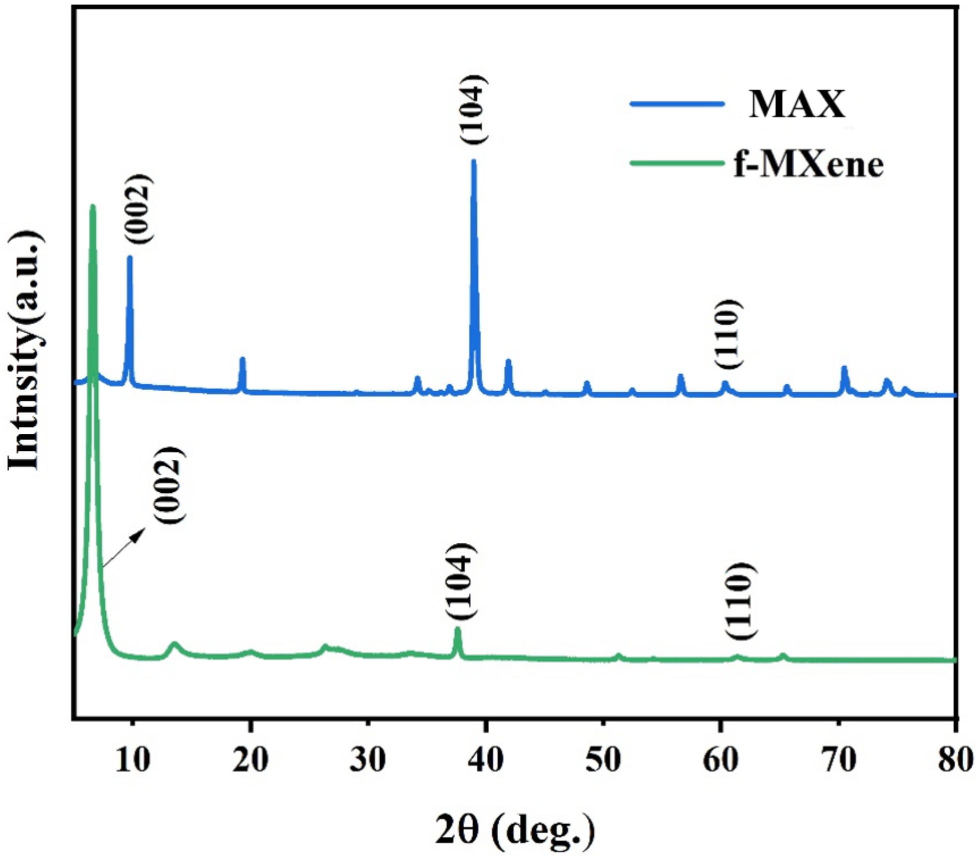

Figure S1. XRD patterns of MAX and f-MXene.

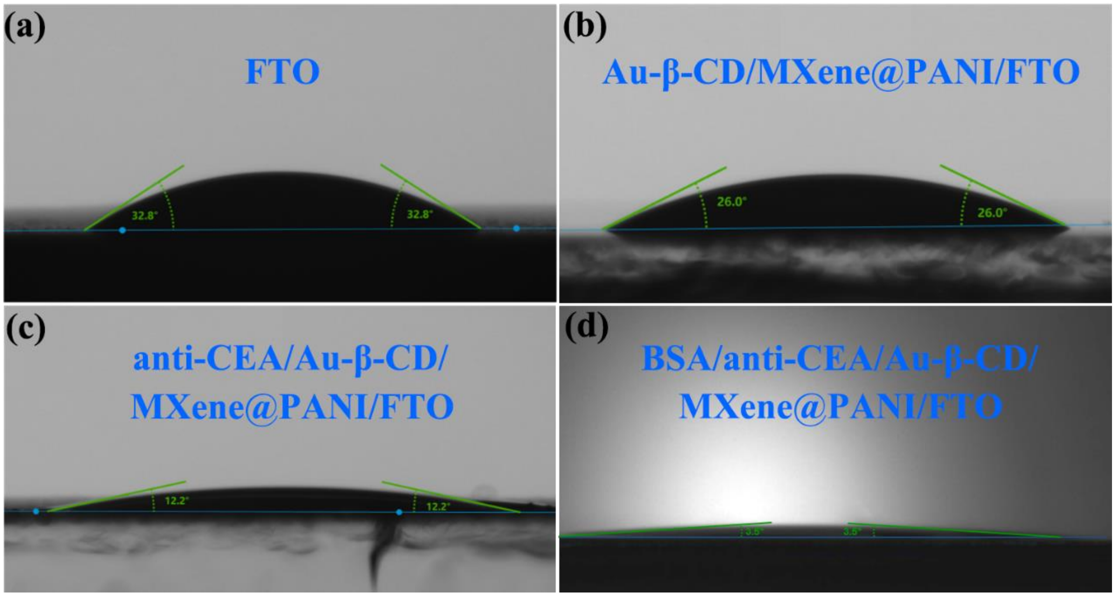

Figure S2. Water contact angle of different electrode (a)FTO, (b)Au-β-CD/MXene@PANI/FTO, (c) anti-CEA/Au-β-CD/MXene@PANI/FTO and (d) BSA/anti-CEA/Au-β-CD/MXene@PANI/FTO.

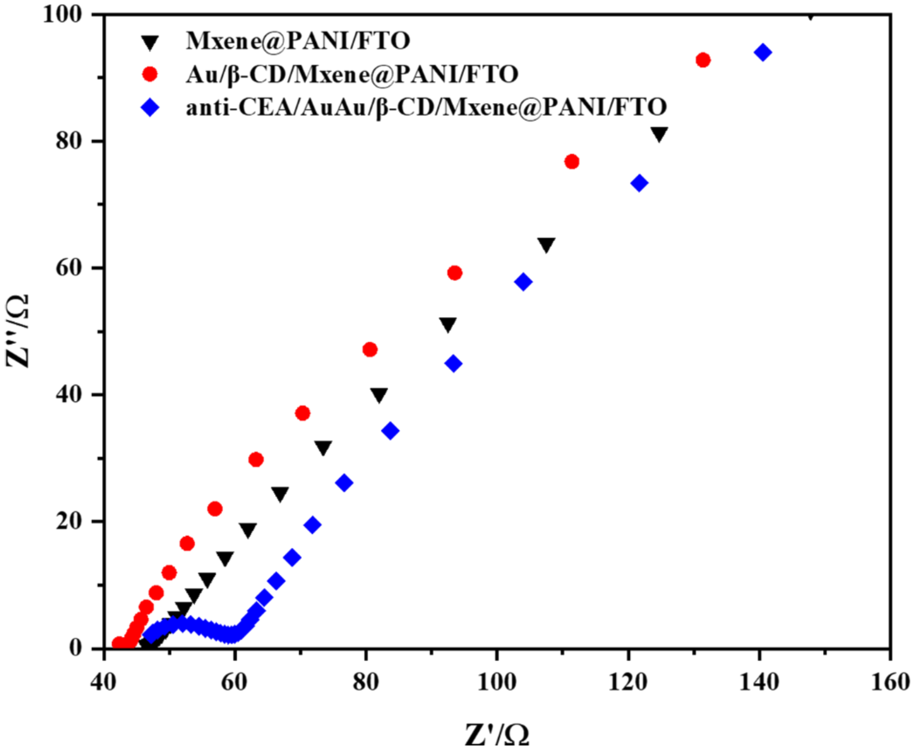

Figure S3. Amplification of electrochemical impedance curves of different modified electrodes.

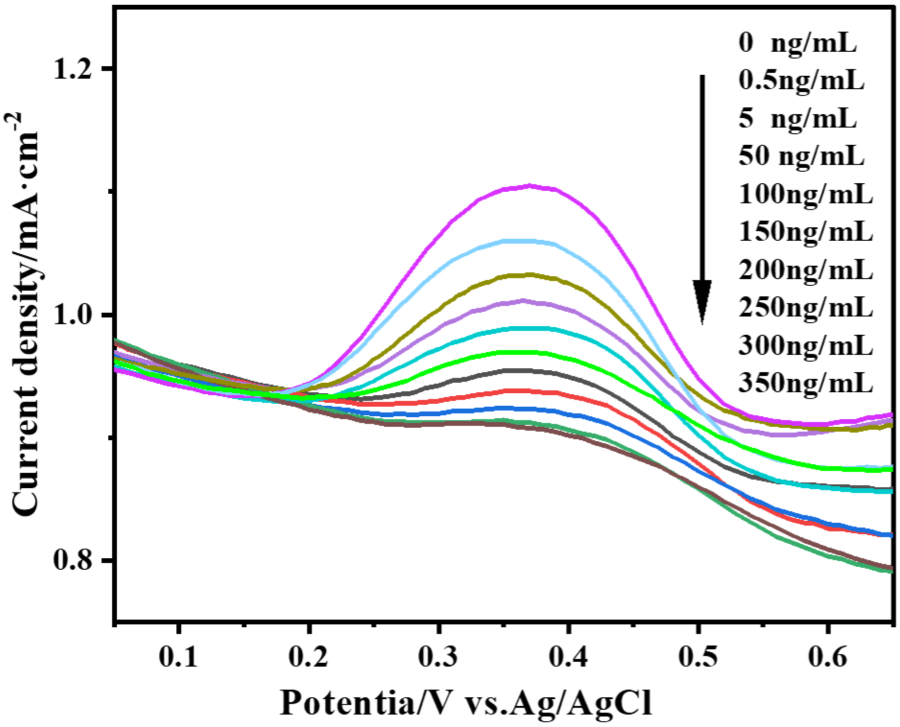

Figure S4. DPV responses for label free immunosensor to CEA at the concentration of 0, 0.5, 5, 50, 150, 200, 250, 300, 350 ng/mL in 0.01M PBS containing 5.0 mM $[Fe(CN)_6]^{3-/4-}$.
